# Air pollution in Boston bars before and after a smoking ban

**DOI:** 10.1186/1471-2458-6-266

**Published:** 2006-10-27

**Authors:** James L Repace, James N Hyde, Doug Brugge

**Affiliations:** 1 Department of Public Health and Family Medicine, Tufts University School of Medicine, 136 Harrison Ave.; Boston, MA 02111, USA; 2 Repace Associates, 101 Felicia Lane, Bowie, MD 20720, USA

## Abstract

**Background:**

We quantified the air quality benefits of a smoke-free workplace law in Boston Massachusetts, U.S.A., by measuring air pollution from secondhand smoke (SHS) in 7 pubs before and after the law, comparing actual ventilation practices to engineering society (ASHRAE) recommendations, and assessing SHS levels using health and comfort indices.

**Methods:**

We performed real-time measurements of respirable particle (RSP) air pollution and particulate polycyclic aromatic hydrocarbons (PPAH), in 7 pubs and outdoors in a model-based design yielding air exchange rates for RSP removal. We also assessed ventilation rates from carbon dioxide concentrations. We compared RSP air pollution to the federal Air Quality Index (AQI) and the National Ambient Air Quality Standard (NAAQS) to assess health risks, and assessed odor and irritation levels using published SHS-RSP thresholds.

**Results:**

Pre-smoking-ban RSP levels in 6 pubs (one pub with a non-SHS air quality problem was excluded) averaged 179 μg/m^3^, 23 times higher than post-ban levels, which averaged 7.7 μg/m^3^, exceeding the NAAQS for fine particle pollution (PM_2.5_) by nearly 4-fold. Pre-smoking ban levels of fine particle air pollution in all 7 of the pubs were in the Unhealthy to Hazardous range of the AQI. In the same 6 pubs, pre-ban indoor carcinogenic PPAH averaged 61.7 ng/m^3^, nearly 10 times higher than post-ban levels of 6.32 ng/m^3^. Post-ban particulate air pollution levels were in the Good AQI range, except for 1 venue with a defective gas-fired deep-fat fryer, while post-ban carcinogen levels in all 7 pubs were lower than outdoors.

**Conclusion:**

During smoking, although pub ventilation rates per occupant were within ASHRAE design parameters for the control of carbon dioxide levels for the number of occupants present, they failed to control SHS carcinogens or RSP. Nonsmokers' SHS odor and irritation sensory thresholds were massively exceeded. Post-ban air pollution measurements showed 90% to 95% reductions in PPAH and RSP respectively, differing little from outdoor concentrations. Ventilation failed to control SHS, leading to increased risk of the diseases of air pollution for nonsmoking workers and patrons. Boston's smoking ban eliminated this risk.

## Background

Secondhand smoke (SHS) has been condemned as a health hazard by all U.S. environmental health, occupational health, and public health authorities [1-7]. This hazard is due to the emission of toxins and carcinogens into indoor air from burning cigarettes, pipes, and cigars, as well as exhaled tobacco smoke from smokers. SHS contains about 4000 chemical compounds, including known carcinogens such as polycyclic aromatic hydrocarbons (PAH), aromatic amines, volatile- and tobacco-specific nitrosamines, as well as a variety of other toxic or irritating compounds, including carbon monoxide, benzene, formaldehyde, hydrogen cyanide, ammonia, formic acid, nicotine, nitrogen oxides, acrolein, and respirable particulate matter [8]. SHS contains 5 regulated hazardous air pollutants, 47 hazardous wastes, and at least 172 chemical toxins [9]. Despite its known hazards, SHS remains a common indoor air pollutant, especially in the hospitality industry, which has had a long history of opposition to efforts to eliminate SHS exposure in restaurants, bars, nightclubs, and casinos.

This report presents the results of air quality monitoring for two SHS marker compounds: respirable particles (RSP) and particle-bound PAH (PPAH) in 7 hospitality venues in the City of Boston, Massachusetts, before and after the city's May 5^th^, 2003 smoking ban. These marker compounds are also harmful air pollutants. A large body of epidemiologic literature associates increases in outdoor air fine particle pollution with increases in acute and chronic mortality, and vice versa. More than 100 studies published over the past 10 years consistently show statistically significant associations between levels of total and cardiovascular mortality and combustion-related outdoor RSP concentrations, and the similarity of pathophysiological mechanisms for RSP exposure from SHS and from outdoor RSP has been noted [10]. Polycyclic aromatic hydrocarbons (PAH) are carcinogens are found in tobacco smoke, and polluted environments such as iron and steel foundries, where such exposures are thought to be the cause of excess cancers in workers. Benzo [a]pyrene (BaP) is the best known PPAH. PAH are potent locally acting carcinogens in laboratory animals inducing lung and upper respiratory cancers of the upper respiratory tract and lung when inhaled, and tumors of the digestive tract when ingested. IARC has concluded that exposure to SHS is carcinogenic to humans [11].

All monitored venues were mechanically-ventilated bars or bar/restaurants, as described in Table 1. The aims of this study were: first, to measure the level of markers for SHS pollution in the hospitality industry of a major American city before and after a smoking ban, so as to assess the contribution of SHS to the fine particle and carcinogen air pollution exposure of restaurant and bar staff and patrons, second, to compare RSP levels to the short-term Federal Air Quality Index and long-term NAAQS to assess acute and chronic health risks, and third, to evaluate the odor and irritation levels from such exposure. Boston passed a Clean Indoor Air Regulation banning workplace smoking in 2003. The study design is model-based, in order to relate observed concentrations to smoker density and air exchange rates for generalizability and comparison to other similar studies [12].

## Methods

### Air quality monitors

In order to assess indoor and outdoor air quality, two fractions of the particulate phase of secondhand smoke were chosen for measurement: respirable particles (RSP), consisting of airborne particulate matter in the combustion size range below 3.5 microns in diameter (PM_3.5_), and particulate polycyclic aromatic hydrocarbons (PPAH). RSP was recorded using a pump-driven ThermoMIE personalDataRAM model pDR-1200 real-time aerosol monitor (ThermoAndersen, Inc., Smyrna, GA), and PPAH was sampled using a pump-driven EcoChem PAS2000CE real-time particle-bound polycyclic aromatic hydrocarbon monitor (EcoChem Analytics, Inc., League City, TX). The pDR1200 was used with a factory calibration of 1.00; the instrument was HEPA-zeroed and the calibration rechecked prior to each day's sampling. PM_3.5 _and PM_2.5_, a regulated outdoor air pollutant, are essentially the same when measuring both the fresh and aged SHS aerosol as essentially the entire SHS distribution is below 1 μm in diameter. The PAS2000CE was also used as factory calibrated. As described in detail elsewhere [12], our pDR 1200's calibration was previously checked against SHS and background aerosol in a series of controlled experiments using 7 Marlboro cigarettes and found to be accurate to within experimental error against both a piezobalance and pump and filter, and simultaneously, our PAS2000ce was evaluated in the same experiment to ascertain the PPAH-to-SHS-RSP ratio. Both devices incorporate data loggers and can output mass concentration and time to a computer; both were synchronized and set for 1-minute averaging times.

### Ventilation assessment

In order to assess ventilation, two methods were used: the first method involved measuring carbon dioxide (CO_2_) using a Langan T15 Personal Exposure Measurer (Langan Instruments, San Francisco, CA), which measures concentrations in real time. Calibration of these MIE and PAS instruments is described elsewhere [12]. If the number of persons in the establishment is counted, the ventilation rate per occupant can be estimated from the difference between the indoor and outdoor CO_2 _levels by using an equation given by The American Society of Heating Refrigerating, and Air Conditioning Engineers (ASHRAE) in ASHRAE Standard 62–1999 [13]. This method is based on carbon dioxide levels in exhaled breath, which will build up in an indoor environment limited only by the ventilation rate. The ventilation rate per occupant defines the rate of supply of outdoor air per occupant of the space, and does not directly measure the rate of pollutant removal. This commonly-used method is limited in accuracy by two potential problems: the CO_2 _levels may not be in equilibrium, and it may be difficult to assess the true outdoor background because of emissions of CO_2 _from nearby traffic.

Accordingly a second method was used to assess ventilation, the air exchange rate method, which relies upon the mass-balance model [14,15]. The air exchange rate is defined as the rate of replacement of polluted air with unpolluted air, and is an index of how fast the secondhand smoke is removed by the air handling system plus sorption on room surfaces. These are described in more detail below.

### Pre-smoking-ban survey methods

The first monitoring phase was conducted on Friday evening, April 18, 2003, prior to enactment of the May 5^th ^smoke-free law in the city of Boston. The criteria for eligibility in the first phase were the presence of visible smoking, that each establishment be within walking distance of the previous, and establishments represent a broad variety of hospitality venues, ranging from a neighborhood bar serving food to a tourist bar serving raw shellfish. Two bar/restaurant venues on the list of candidates were rejected because no-one could be found smoking at entry, and time was limited by PPAH monitor battery charge. The venues were selected by one of us (JH) a Boston resident, who identified the venues to be sampled.

Venues were visited for an average of about 36 minutes (range, 20 to 59 min). Outdoor and in-transit locations were sampled before and after each venue, as well as a nonsmoking hotel room before and after the pub survey. The miniaturized monitors were concealed in wheeled luggage, and sampling was discreet in order not to disturb occupants' normal behavior. All venues were well-patronized during the measurements. The monitoring package was generally unobtrusively located along a wall, or beneath a table, ~2 ft from the floor.

Each pub's dimensions were measured using a Calculated Industries Dimension Master ultrasonic digital ruler (range 2 ft – 50 ft, resolution ± 1%), by a Bushnell Yardage Pro Sport Compact infrared laser Rangefinder (range 10 yd to 700 yd, resolution ± 1 yd), or estimated by pacing, if the venue was too crowded or irregular in shape. The total number of persons and the number of burning cigarettes was counted every ten minutes, including the beginning and end of the sampling period. The clock time upon entering and leaving each establishment was recorded in a time-activity pattern diary, so that each venue's concentration could be identified by time recorded in the data.

### Post-smoking-ban survey methods

The second monitoring phase was conducted six months later, on Friday evening, October 17, 2003, after compliance with the law had been amply demonstrated, and the temperature was sufficiently cool such that the venues were not open to the outdoor air and the baseline indoor air quality could be assessed in the absence of smoking. Eligibility criteria were as in Survey #1, except that in all venues no smoking was observed. The same 7 hospitality venues were visited for an average of about 43 minutes (range, 21 to 71 min), after the smoking ban took effect, and it was judged that their compliance with the ban was satisfactory. Continuous measurements of RSP and PPAH, were again made from ~6 PM to 12 AM, in the same order and at about the same time of night. As in the pre-ban field study, control measurements were performed outdoors, in transit, and in a non-smoking room on the same floor at the same hotel.

### SHS odor and irritation

Odor and irritation thresholds have implications for smoking policy development. Weber and Grandjean [25] report that nearly three-fourths of nonsmokers were disturbed by smoky air in restaurants, that acute irritation from SHS is enhanced in warm and dry air, and that controlled studies of healthy nonsmokers show that the particulate phase of SHS is mostly responsible for the irritating effects of SHS, while the gas phase is responsible for most of the annoyance. Weber and Grandjean [25] also found that irritation, as measured by eye-blink rate, increased linearly with increasing smoke concentration, and with increased duration of exposure at a constant concentration. The same results were observed, although less pronounced, for nose and throat irritations. Unlike irritation, annoyance increases rapidly as exposure begins, then plateaus with time.

Junker et al. [26], conducted a study of 24 healthy nonsmokers aimed at determining air quality standards required to protect nonsmokers from adverse health effects caused by impacts of SHS from smoldering cigarettes on the human sensory system as well as to provide measures for establishing acceptable indoor air quality. Junker et al. [26] found that that the threshold for objectively measured sensory irritation was about 4.4 μg/m^3 ^for PM_2.25_, and that at this level, 67% of the nonsmoking subjects judged the quality of the air to be unacceptable. In addition, Junker et al. [26] measured a median odor-detection threshold of about 1 μg/m^3 ^SHS-PM_2.25_. These authors concluded that the results for sensory symptoms show that even at very low SHS concentrations, subjects perceived a significant increase in sensory impact (eye, nasal, and throat irritation), and felt significantly more annoyed and reported the quality of the air to be less acceptable than exposure to zero levels of SHS.

### The active smoker model

The model-based study design allows the data to be generalized: in the April 18^th ^survey, values for area, volume, active smoker count, and pollutant concentration were measured. From these values the smoker density can be computed, and air exchange rate due to ventilation can be estimated using a simplified version of the mass-balance model called the Active Smoker Model (Eq. 1 below) [12]. This equation calculates, in units of micrograms of pollutant per cubic meter of air (μg/m^3^), the level of uniformly-mixed time-averaged SHS-RSP in a building as a function of the active smoker density D_s_, in units of burning cigarettes per hundred cubic meters (BC/100 m^3^) and the building's air exchange rate C_v_, in units of air changes per hour (h^-1^):

RSPETS=650DsCv     (Eq. 1),
 MathType@MTEF@5@5@+=feaafiart1ev1aaatCvAUfKttLearuWrP9MDH5MBPbIqV92AaeXatLxBI9gBaebbnrfifHhDYfgasaacH8akY=wiFfYdH8Gipec8Eeeu0xXdbba9frFj0=OqFfea0dXdd9vqai=hGuQ8kuc9pgc9s8qqaq=dirpe0xb9q8qiLsFr0=vr0=vr0dc8meaabaqaciaacaGaaeqabaqabeGadaaakeaacqWGsbGucqWGtbWucqWGqbaudaWgaaWcbaGaemyrauKaemivaqLaem4uamfabeaakiabg2da9iabiAda2iabiwda1iabicdaWmaalaaabaGaemiraq0aaSbaaSqaaiabdohaZbqabaaakeaacqWGdbWqdaWgaaWcbaGaemODayhabeaaaaGccaWLjaGaaCzcamaabmaabaacbeGae8xrauKae8xCaeNae8Nla4IaeeiiaaIae8xmaedacaGLOaGaayzkaaGae8hlaWcaaa@45F9@

The relationship of the number of burning cigarettes to the number of smokers present is illustrated as follows: the 2003 Massachusetts average adult habitual smoking prevalence is 19.7% (± 1%) [24]. Thus in a group of adult Bostonians consisting of mixed smokers and nonsmokers according to the Statewide smoking prevalence, 19.7% of the entire group would be expected to be habitual smokers. Of those, 1/3, or ~6.6% would be expected to be observed actively smoking at any one time [12]. Thus in a 2003 field survey of a venue in Boston, the prevalence of active smoking would be expected to be 6.6% of persons present if the smoking prevalence is representative of that in the larger state population. Table 2 shows that the mean active smoking prevalence actually observed in the pre-ban survey is about 2/3 of this value, at 4.04% (SD 1.6%) for all 7 venues sampled. This may reflect a lower smoking prevalence among affluent urban Bostonians than in the rest of the State.

For a bar with a percentage of smokers equal to the 2003 Massachusetts smoking prevalence rate of 19.7% [33], at maximum occupancy, the default smoker density is (0.197 smokers/occ)(100 occ/10,000 ft^3^) = 19.7 smokers per 10,000 ft^3^, or in metric units, 19.7 smokers per 283 cubic meters (m^3^), of whom 1/3 would be expected to be actively smoking at any one time yielding an estimated active smoker density of D_s _= (1/3)(19.7)/2.83 = 2.32 active smokers (i.e., burning cigarettes (BC) per 100 m^3^. Using Eq. 1, the expected SHS-RSP concentration for a properly ventilated Boston bar at maximum occupancy is: SHS-RSP = 650(2.32)/18 = 83 μg/m^3 ^above background. Note that if the SHS-RSP concentration and smoker density are measured, the air exchange rate for SHS-RSP removal can be calculated. Note that the model implicitly assumes a default surface decay rate for RSP = 1.33 C_v _[9].

### Ventilation rates per occupant from CO_2_

CO_2 _is a waste product of human metabolism, and will buildup in the air proportionally to the number of persons in the building environment. Accordingly, ventilation systems are designed with CO_2 _control in mind. The design ventilation engineer's guideline for ventilation rates in buildings is ASHRAE Standard 62–1999 [13]. Equation 2 is typically used by engineers to estimate the ventilation adequacy based upon an indoor CO_2 _measurement. Eq. 2 is given in Appendix C of ASHRAE Standard 62 [13], and specifies the estimation of *C*_s_, the equilibrium CO_2 _levels in parts per million (ppm) in a venue:

Cs=NVo+Co     (Eq. 2),
 MathType@MTEF@5@5@+=feaafiart1ev1aaatCvAUfKttLearuWrP9MDH5MBPbIqV92AaeXatLxBI9gBaebbnrfifHhDYfgasaacH8akY=wiFfYdH8Gipec8Eeeu0xXdbba9frFj0=OqFfea0dXdd9vqai=hGuQ8kuc9pgc9s8qqaq=dirpe0xb9q8qiLsFr0=vr0=vr0dc8meaabaqaciaacaGaaeqabaqabeGadaaakeaacqWGdbWqdaWgaaWcbaGaem4Camhabeaakiabg2da9maalaaabaGaemOta4eabaGaemOvay1aaSbaaSqaaiabd+gaVbqabaaaaOGaey4kaSIaem4qam0aaSbaaSqaaiabd+gaVbqabaGccaWLjaGaaCzcamaabmaabaacbeGae8xrauKae8xCaeNae8Nla4IaeeiiaaIae8NmaidacaGLOaGaayzkaaGae8hlaWcaaa@40B6@

where *N *is the CO_2 _generation rate per person (N = 0.30 L/min, or 5000 ppm-L/s-occupant corresponding to office work), *V*_o _is the outdoor air flow rate per occupant in L/s, and *C*_o _is the CO_2 _concentration (expressed in parts per million or ppm) in the outdoor air.

The CO_2 _levels measured in this survey are given in Table 2, and used to calculate *V*_*o *_in the right-most column of table 2. The ASHRAE Standard recommended value for *V*_*o *_is 15 L/s-occ at maximum occupancy, essentially to control human bioeffluents. CO_2 _concentrations in acceptable outdoor air typically range from 300 ppm to 500 ppm, and maintaining a level of 15 L/s-occ should result in a steady-state CO_2 _concentration of about 350 ppm above background. Thus expected CO_2 _concentrations for a venue in compliance with ASHRAE Standard 62 should result in a concentration of the order of 850 ppm or less, and levels above 1000 ppm are consistent with poor ventilation. Note that the air exchange rate calculated from the model refers to the removal of SHS by ventilation and surface decay, while the CO_2 _calculation refers to human bioeffluent removal.

## Results

### Pre-ban

Weather conditions measured at Logan Airport on the Harbor on Friday evening April 18, 2003 (6 PM to Midnight) were fair and cold, with barometric pressure between 30.57 and 30.54 inches of mercury. The outdoor temperature was 5°C (41°F) at 6 PM, decreasing to 4°C (39°F) by Midnight. Outdoor relative humidity ranged from 76% to 87% during the same hours [16]. However, the environmental parameters inside the monitoring package were measured using the Langan Personal Exposure measurer, which was deployed in the Downtown Boston area during this survey, were less extreme, with temperature varying from 12.7°C to 20.9°C, with a mean 17.3°C, and relative humidity ranging from 25% to 64%, with a mean of 43.5%.

**Figure 1:**
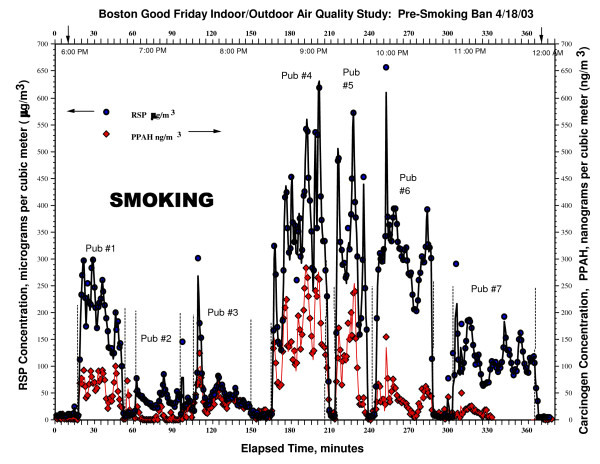
Measurements of respirable particle (RSP) and carcinogen pollution (PPAH) as a function of time before the Boston smoking ban on Friday, April 18, 2003 from 6 PM to 12 AM in 7 hospitality venues. Outdoor levels are indicated between the dotted lines showing the levels in each pub. Contrast with Figure 2.

Table 2 organizes the April 18 pre-smoking-ban study results. The April 18 RSP and PPAH data are plotted in Figure 1. Figure 1 shows a characteristic pattern of low outdoor RSP and PPAH levels, with indoor RSP and PPAH levels in all pubs quite elevated with respect to the outdoors. Pub # 6 has a carbon monoxide (CO) level twice as high as the other pubs, whose CO levels on average are comparable to outdoors. Figure 3 shows a plot of the RSP levels vs. the PPAH levels, excluding Pub #6 [which had an indoor air quality problem unrelated to smoking as discussed below]. Figure 3 shows a linear relationship (R = 0.93) between RSP and PPAH in the pubs suggesting that the PPAH carcinogens are due to SHS, as found in controlled experiments which show that SHS-PPAH levels track the SHS-RSP levels, and that both are elevated during smoking and decay toward background levels when the cigarettes are extinguished [12].

Excluding Pub #6, the indoor levels of RSP average 179 μg/m^3^, ~10 times higher than the outdoor RSP levels, which averaged 18.6 μg/m^3^, and ~28 times higher than in the hotel room, where measurements were taken in front of an open window. Similarly, the PPAH levels, again excluding Pub #6, average 65.1 ng/m^3 ^in the pubs, ~4 times higher than the outdoor levels, which averaged 15.8 μg/m^3^, and 23 times higher than the hotel room.

### Post-ban

The same venues were sampled on Friday evening October 17, 2003 (6 PM to Midnight) at the same time of night as in the pre-ban survey. Weather (6 PM to Midnight) was overcast and mild, with barometric pressure between 30.09 inches of mercury to 30.12 inches of mercury. The outdoor temperature was 48.2°F (9°C) at 6 PM, increasing to 50.0°F (10°C) by midnight. Relative humidity ranged from 58% to 62% during the same period [16].

**Figure 2:**
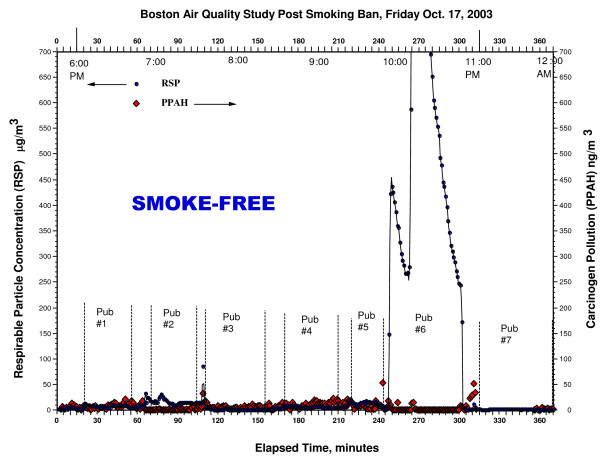
Measurements of RSP and PPAH as a function of time after the Boston smoking ban on Friday, October 17, 2003 from 6 PM to 12 AM in the same 7 hospitality venues shown in Figure 1. Pub #6 had high carbon monoxide levels before and after the ban; this was reported to Boston Public Health, whose investigation later disclosed this was due to fumes from a malfunctioning gas-fired deep fat fryer. Outdoor air pollution levels appear between the dotted lines bracketing the indoor levels in each pub.

Table 3 organizes the Oct. 17 post-ban study results. Zero smokers were observed in all pubs post-ban. The Oct. 17 RSP and PPAH data are plotted in Figure 2. Figure 2 shows a characteristic pattern of low indoor and outdoor RSP and PPAH levels, except for the anomalous RSP levels in Pub # 6. Pub # 6 results show that the RSP is more than an order of magnitude greater than for any other pub, while the PPAH levels are the lowest of any pub. This indicates that the smoking created the elevated PPAH levels shown in Figure 1 for Pub # 6, but that there is another source for the RSP. As in Table 2, Table 3 shows that Pub # 6 also has an elevated carbon monoxide (CO) level, 6 times that of the mean for the other pubs, which again have CO levels on average comparable to outdoors. Again excluding Pub #6, the indoor levels of RSP average 7.73 μg/m^3^, ~99% of the outdoor RSP levels, which averaged 7.82 μg/m^3^, and only ~4 times higher than in the hotel room. Similarly, the PPAH levels, again excluding Pub #6, average 5.64 ng/m^3 ^in the pubs, ~62% of the outdoor levels, which averaged 9.05 ng/m^3^, and 2.2 times higher than the hotel room. The hotel room RSP levels were 3 times higher on April 18 than on Oct. 17, but still relatively low, on both surveys, and PPAH levels were essentially the same on both occasions.

### Odor and irritation results

In table 5, the SHS-RSP values for the most-polluted venue, Pub #4 exceed Junkers' irritation threshold by a factor of (332)/4.4 = 75-fold, and exceed Junkers' odor threshold [26] by a factor of 332. For the least SHS-polluted venue, Pub # 3, the irritation and odor ratios are still 13 times and 57 times the threshold levels. For all venues averaged, these thresholds are exceeded by factors of 39 to 171 respectively. The lack of an adverse economic impact in the hospitality industry due to Massachusetts' smoke-free workplace law one year [17] later may be due in part to the reductions in odor and irritation from SHS, making these venues more attractive to nonsmokers [29].

## Discussion

### Smoker density

The observed smoker density ranges from 0.03 BC/100 m^3 ^to 1.31 BC/100 m^3^, and averages 0.57 BC/100 m^3^, just 25% of the 2.32 BC/100 m^3 ^expected at maximum occupancy.

### Air exchange rates from the model

The default air exchange rate for a typical bar at maximum occupancy was derived by Repace [12] as *C*_*v *_= 18 air changes per hour (h^-1^). Using Eq. 1, *C*_*v *_is calculated for all 7 venues in Table 2, ranging from *C*_*v *_= 0.75 to 4.23 h^-1^, also much lower than expected, indicating these bars are underventilated.

### Ventilation rates from CO_2_

Calculated *V*_*o *_values in Table 2 range from 5 to 29 L/s-occ, and average about 14 L/s-occ, close to the 15 L/s-occ specified by ASHRAE. However, the mean occupancy was 39 occupants per 1000 ft^2^, 39% of maximum occupancy for a bar, indicating that air quality would be much worse at busier times. This illustrates even if the ventilation rate for removal of CO_2 _is adequate, the air exchange rate for SHS removal can be inadequate because *V*_*o *_is not coupled to smoker density. It also illustrates that at full occupancy, none of the venues would have complied with ASHRAE Standards, showing that proper ventilation has been ignored in these venues.

### Air pollution from SHS

**Figure 3:**
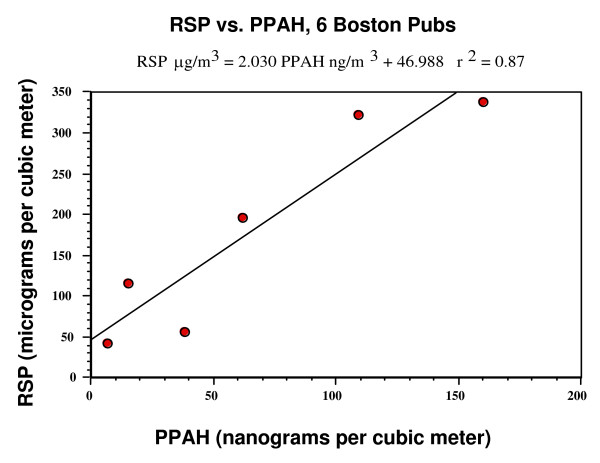
The regression of respirable particle pollution against carcinogen pollution in 6 of 7 Boston pubs studied before the smoking ban. Pub # 6 is excluded due to apparent contamination from kitchen fumes. The ratio for RSP/PPAH in the same units is about 2000:1. This is the same RSP/PPAH ratio found in the Wilmingon, Delaware study (Repace, 2004).

**Figure 4:**
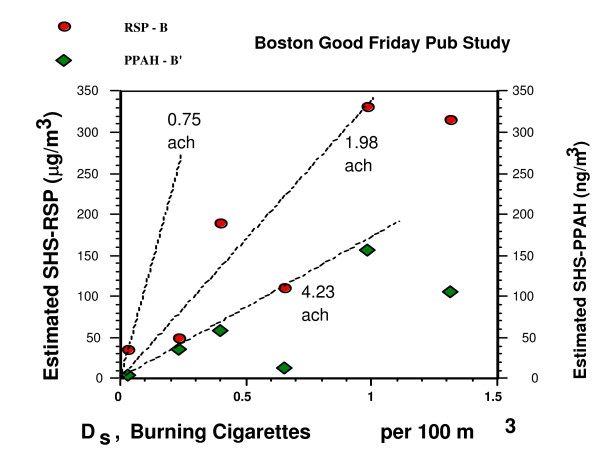
Pre-ban secondhand smoke respirable particulate, SHS-RSP, (total measured RSP – background RSP, B) in micrograms per cubic meter and SHS-PPAH concentration (total measured PPAH – background PPAH, B') in nanograms per cubic meter versus burning cigarette density D_s _(active smokers observed per hundred cubic meters of space volume) and air exchange rate C_v _in units of air changes per hour (ach) as calculated from RSP using the model of Repace (2005). The decay rates of PPAH are higher than for RSP. Background-subtraction values are arbitrarily chosen from measured open-window nonsmoking hotel room values. Data from Pub # 6 are omitted from this plot.

Figure 3 plots the pre-ban RSP vs. the pre-ban PPAH. A regression analysis yields a good linear fit (R = 0.93) with a 2000:1 ratio between RSP and PPAH. This is in good qualitative agreement with previous research which shows that during smoking, the cigarette PPAH tracks the RSP, but has a higher decay rate [12]. Figure 4 plots the background-subtracted RSP vs. the background-subtracted PPAH values as a function of burning cigarette density and SHS-RSP air exchange rate using the habitual smoker model. The correlation of net RSP and net PPAH with each other and the increase of PPAH and RSP with active smoker density suggest a strong association with smoking, and interestingly, the slope of the regression differs only by 1% from that observed in the Wilmington Study [12].

By how much are the RSP and PPAH levels reduced by the smoking ban? From Table 2, excluding Pub # 6, which had the IAQ problem, the pre-ban pub RSP levels average 179 μg/m^3^. From Table 3, the post-ban pub RSP levels, again excluding Pub #6, average 7.7 μg/m^3^, a decrease by 96%. Similarly, From Table 2, excluding Pub #6, the pre-ban pub PPAH levels average 65.1 ng/m^3^. From Table 3, the post-ban pub PPAH levels, again excluding Pub #6, average 6.32 ng/m^3^, a decrease by 90%. If the calculations are referenced to the indoor/outdoor levels on April 18, the estimated SHS-RSP contribution is [(179-18.6)/179] = 90%, and the estimated SHS-PPAH level contribution is [(65.1-15.8)/65.1] = 76%. However the latter calculation may be an underestimate, since the PPAH level in the pubs on Oct. 17, 6.32 ng/m^3^, was about 70% of the outdoor level; if the PPAH outdoor level on April 18 is adjusted downward to 70% of its value (0.70)(15.8) = 11 ng/m^3^, and the estimated SHS-PPAH concentration recalculated, [(65.1-11)/65.1] = is 83%. Thus, a conservative inference from the data would be that SHS contributed about 90% to 95% of the RSP levels during smoking, and 80% to 90% of the PPAH levels during smoking, with an average smoking prevalence of about 12%. This compares to a state-wide smoking prevalence of 19.7% in 1999, as reported above.

But there was one major exception: Pub # 6, which had a higher RSP level after the smoking ban than before (although the PPAH level was much lower). Repace et al. (1980) [14] found that cooking smoke could contribute significantly to indoor air pollution. Kitchens are supposed to remain under negative pressure to contain cooking fumes [36]. However, Table 2 shows that Pub #6's CO level on April 18 was [(5.5-2.16)/(0.38)] = 8.8 standard deviations beyond the mean of the other pubs. Similarly, Table 3 shows that Pub #6's CO level on Oct. 17 was also high, at [(7.94-1.24)/(0.85)] = 7.9 standard deviations beyond the mean of the others. This suggests that Pub # 6 had an indoor air quality problem of another type. The Boston Public Health Commission (BPHC) was alerted, and conducted an investigation. The investigation discovered that a gas-fired deep-fat fryer had a yellowish flame instead of the expected blue, as a result of the burner being plugged with grease. These yellow flames emitted 50 ppm of CO into the kitchen, which permeated the rest of the premises, although the kitchen exhaust hoods were functioning (L. Bethune, Boston Public Health Commission, Office of Environmental Health, personal communication).

How do these air quality measurements compare with other studies? In a preliminary report on a similar model-based RSP study in 27 Boston hospitality venues with smoking, but with both pre- and post-ban data taken using an aerosol monitor, Connolly et al. (2005) [17] in a Harvard study, reported a mean estimated SHS-RSP of 207 μg/m^3 ^(SD 202), and a median value of 121 μg/m^3^. Connolly et al.'s smoker density *D*_*s *_varied between 0 and 2.95 BC/100 m^3^, with a mean value of 0.89 (SD 0.73) compared to 0.57 (SD 0.44) in our study. Our mean pre-ban estimated SHS-RSP is (198 – 19) = 179 μg/m^3 ^(Table 2), and a median value of 178 μg/m^3 ^(not shown), and a mean estimated SHS-PPAH level of (61.7-15.8) = 46 ng/m^3^.

In a very similar model-based air quality survey to that reported here, Repace [12] measured RSP and PPAH in Wilmington, DE in 8 hospitality venues, a casino, 6 pubs, and a pool-hall. In the Wilmington study, active smoker density varied between 0.02 and 1.44 cigarettes per hundred cubic meters and averaged 0.53 (SD 0.54), and SHS contributed 90% to 95% of the RSP air pollution during smoking, and 85% to 95% of the carcinogenic PPAH, with an average smoking prevalence of 15%. Indoor RSP levels averaged 231 μg/m^3 ^(SD 207), quite similar to the values reported by the Massachusetts Study [17]. Ott [38], in a model-base study, observed reductions of RSP 84% following California's smoking ban, in a 2-year longitudal study in a tavern in California, and reported that the active smoking count explained more than 50% of the variation in the RSP concentrations observed on individual visits [38].

Another model-based survey in Western New York State reported a range in smoker density in 14 bars and restaurant/bars from 0.25 to 3.15 BC/100 m^3^, averaging 1.36 BC/100 m^3^; the mean estimate SHS-RSP level was 385 μg/m^3^, and the total RSP pollution level declined by 93% after a state-wide smoking ban [39]. This is in good agreement with a study of the effectiveness of a state-wide smoking ban in New York State, where urine cotinine levels, a measure of SHS exposure, declined by 94%, from a pre-ban median of 4.93 ng/ml in non-casino hospitality workers (n = 36) to a post-ban level of 0.3 ng/ml (n = 27), the level of detection [30].

Similar results have been observed in Europe. Mulcahy et al. [23] randomly sampled 20 city centre bars in Galway, Ireland, for air nicotine concentrations before and after the Irish national smoking ban. They found an 83% reduction in air nicotine concentrations following the smoking ban. However, smoker density was not reported. Edwards et al. [37] conducted a cross sectional study in four mainly urban areas of the North West of England measuring a mean PM_2.5 _level of 285.5 μg/m^3 ^(95% CI 212.7 to 358.3), in a stratified random sample of 64 pubs; smoker density was not reported. Levels were higher in pubs in deprived communities: mean 383.6 μg/m^3 ^(95% CI 249.2 to 518.0) vs 187.4 μg/m^3 ^(144.8 to 229.9). The highest outdoor levels observed were about 24 μg/m^3 ^suggesting that overall, about 92% of the RSP levels might have been due to SHS. The UK will ban smoking in pubs in 2007.

The Boston pre-ban PPAH results 61.7 ng/m^3 ^(SD 54.9), half of those found in the Wilmington air quality study, 134 ng/m^3 ^(SD 86.5), whereas the smoker density varied from 0.03 to 1.31 in Boston, and averaged 0.57 (SD 0.44). However the average air exchange rate in the Boston study was higher, at 2.26 h^-1 ^(SD 1.37), compared to 1.4 h^-1 ^(SD 0.97) in the Wilmington Study. Further, to place the preban Boston PPAH results into perspective, they are compared with PPAH measurements in outdoor air measured in nine sites in Roxbury, a Boston neighborhood polluted by heavy diesel bus and truck emissions. Median Roxbury concentrations ranged from 4 to 57 ng per cubic meter (ng/m^3^), and averaged 18 ng/m^3 ^over all sites [18]. Our PPAH levels average (61.7/18) = 3.4 times as high as on the most heavily travelled roadways in Boston. Finally, a regression of the SHS RSP vs. SHS PPAH yields a ratio of ~2030:1, in excellent agreement with the value of 2054:1 reported in the Delaware study [12].

### Air quality and health

To place the predicted and observed levels of RSP into perspective, consider the U.S. Annual National Ambient Air Quality Standard (NAAQS) for particulate matter 2.5 microns in diameter or less (PM_2.5_), which encompasses combustion-related fine particulate by-products such as tobacco smoke, chimney smoke, and diesel exhaust. In 1997, the EPA promulgated a 24-hour NAAQS for PM_2.5_, of 65 μg/m^3^, not to be exceeded more than once per year, and an annual NAAQS for PM_2.5 _of 15 μg/m^3^, based on protecting human health [19,20,35]. The NAAQS for PM_2.5 _is designed to protect against such respirable particle health effects as premature death, increased hospital admissions, and emergency room visits (primarily the elderly and individuals with cardiopulmonary disease); increased respiratory symptoms and disease (children and individuals with cardiopulmonary disease); decreased lung function (particularly in children and individuals with asthma); and against alterations in lung tissue and structure and in respiratory tract defense mechanisms in all persons. [19]. PM_2.5 _and PM_3.5 _are closely related [21]. The annual average PM_2.5 _level for Boston (City Square) for 2001 was: 13.25 μg/m^3 ^[34]. 90% of U.S. Counties have PM2.5 levels below about 16 μg/m^3 ^[22]. The intent of the NAAQS is to limit risk to human health from exposure to particulate air pollution. The NAAQS does not apply *de jure *to indoor air quality because the U.S. Clean Air Act specifies only outdoor ambient air and as such is not an exposure standard, however, this health-based standard may be used *de facto *to evaluate levels of indoor air quality provided averaging times are taken into account. We did not consider using OSHA workplace standards as a basis of comparison, because they are far less protective of human health than EPA standards. Recent research on the adverse health effects of fine particle pollution shows estimated concentration-response functions that are approximately linear, with no evidence of safe threshold levels; moreover, unresolved gaps in understanding exist concerning who is most at risk or most susceptible [10].

The average pre-ban SHS PM_3.5 _level in the 6 pubs (excluding Pub #6) was 179 μg/m^3^, and post-ban 7.73 μg/m^3^. Subtracting post-ban background, and assuming pub staff work 260 days per year, 8 hrs per day, they are exposed to an annual average of (171 μg/m^3^)(260 d/365 d)(8 hr/24 hr) = 40.6 μg/m^3 ^from SHS, and to an annual average background level of 13.25 μg/m^3 ^from outdoor non-SHS sources. Assuming that these averages are sustained over the required 3 year averaging period, SHS exceeds the 15 μg/m^3 ^level of the Annual National Ambient Air Quality Standard by a factor of (40.6 + 13.25)/15 = 3.6. Although no standards have been set for PPAH, assuming an 8-hr workday, on a 24-hr average basis for the 7 venues sampled, pre-ban PPAH exceeded post-ban PPAH levels by a factor of [(65.1/3) + 6.32)]/6.32 = 4.1, significantly increasing exposure of workers to substances known to be implicated in the causation of cancer, heart disease, and stroke [12,31,32].

Figures 1, 2, and 3 taken together demonstrate conclusively that secondhand smoke causes most or a significant fraction of the massive RSP and PPAH pollution elevations shown in 6 of 7 hospitality venues of Figure 1. Smoking in these Massachusetts hospitality venues caused levels of respirable particles and particle-bound PAH carcinogens exposure to increase by six-to-ten-fold. The models developed from Equation 1 generalize the results to other hospitality venues. Finally, the elevated carbon monoxide levels and heavy RSP pollution in Pub #6 before and after the smoking ban suggest that the kitchen exhaust equipment has broken down and grilling fumes are being pulled into the dining room along with cooking gas fumes from a defective deep-fat fryer. Overall, RSP levels decreased from 179 μg/m^3 ^to 8 μg/m^3^, or by 96% and PPAH levels decreased from 65 ng/m^3 ^to 6 ng/m^3^, or by 90%. There are few public policy interventions that require such a small public investment and that yield such a dramatic return in such a short period of time.

### Health risk assessment for workers and patrons

What are the disease risks of SHS-RSP at the odor and irritation thresholds? Repace et al. estimated [27] that lung cancer and heart disease mortality risk combined from workplace SHS (annualized workplace exposure of 6.7 hours daily) for a working lifetime of 40 years was 150 deaths per million persons at risk per 1 μg/m^3^. Since the federal (EPA) *de minimis *risk level is 1 death per million workers at risk, at the lowest odor threshold ever measured, the risk from passive smoking is 150 times *de minimis *risk, and at the lowest irritation level ever measured, 600 times *de minimis *risk. the *de minimis *risk is defined as a level "below regulatory concern" [28]. In other words, if SHS can be smelled, it's at harmful levels.

At the 179 μg/m^3 ^level SHS-RSP averaged over all venues, the chronic risk of these two diseases combined is (179/1)(150 × 10^-6^) = ~27 deaths per 1000 workers per 40 year working lifetime. This exceeds the Occupational Safety and Health Administration's Significant Risk of Material Impairment of Health level of 1 death per 1000 per 45 years [27] by a factor of (45/40)(27 per 1000)/(1 per 1000) = 30-fold. Thus these exposures were quite significant [28] by U.S. federal risk assessment standards for occupational and environmental health.

Air Quality forecasts are provided by State and local agencies, using the U.S. Environmental Protection Agency's (EPA) Air Quality Index (AQI) [22], a uniform index that provides general information to the public about air quality and associated health effects. These index descriptors are described in Table 4. Health advisories and warnings are based on the current AQI as well as the forecasted AQI. Air quality authorities maintain running averages for each pollutant, and an appropriate AQI is reported that generally corresponds to the current average. For most major cities, air quality forecasts, based on predicted meteorological conditions and monitored air quality, are also released to the public usually during the afternoon hours of the day preceding the forecast period. These forecasts are for PM and ozone, since these are the pollutants that generally contribute to unhealthy air quality. If pollutant levels are expected to be unhealthy, the state and local agencies will release a color-coded health warning or advisory to the local media and post these advisories on their web sites [22]. The color codes and corresponding normalized Air Quality Indices are based upon "break-points" or ranges of minimum-to-maximum particulate levels corresponding to increasing severity of expected health effects. AQI values are usually below 100, with values greater than 100 occurring at most several times a year. The SHS-RSP levels in Table 5 for the 7 pubs range from AQI descriptors corresponding to Unhealthy for Sensitive groups (2), to Unhealthy (1), to Very Unhealthy (1), to Hazardous (3). This comports with Biener et al.'s [29] reported health reasons for nonsmokers' aversion to SHS. The air pollution levels overall correspond for all venues to a level of 198 μg/m^3^, or Very Unhealthy.

## Conclusion

Our air quality survey in 7 Boston Massachusetts pubs indicates that Boston's smoke-free law reduced RSP pollution by 90% to 95% and PPAH pollution by 80% to 90%. Few public investments have yielded such large public health gains in such a short period of time at so little cost. Pre-ban air pollution levels ranged from Unhealthy for Sensitive groups to Hazardous, and on average corresponded to Very Unhealthy levels as judged by the AQI for outdoor PM_2.5_. Post-ban AQIs were in the Good range, except in one pub that had a malfunctioning kitchen appliance. This pub was excluded from the air quality averages. RSP and PPAH levels were correlated during smoking and were proportional to the density of burning cigarettes. While ventilation rates were generally in compliance with design rates at the 39% average occupancy, at maximum occupancies they would not have met ASHRAE Standard 62–2001 recommendations. SHS risk to workers exposed at the 6-pub average exceeds OHSA' Significant Risk level by a factor of 30 for lung cancer and heart disease combined. Workplace exposures to SHS-RSP exceeded the U.S. NAAQS 4-fold. Carcinogenic risk apart, ventilation was incapable of controlling RSP to meet the NAAQS without a 100-fold increase in outdoor air supply. Smoke-polluted pubs had average levels of fine particles and particulate carcinogens which were ten-fold and three-fold higher respectively than previously reported for Boston streets with heavy truck and bus traffic. Averge SHS-RSP values exceeded irritation and odor thresholds by factors of 39 to 171 respectively. Daily SHS-PPAH exposures were quadrupled relative to outdoors. The lack of an economic impact from Massachusetts' smoke-free workplace law may have resulted from reductions in odor and irritation, making hospitality venues more attractive to the nonsmoking majority.

## Clinical significance

Nonsmoking hospitality workers and patrons are exposed to unhealthy levels of air pollution and high levels of irritation and odor from secondhand smoke.

## Competing interests

JN Hyde and D Brugge declare they have no competing interests. JL Repace is a secondhand smoke consultant, and has served as an expert witness in litigation involving secondhand smoke morbidity and mortality.

## Authors' contributions

JLR, JNH, and DB conceived the study and participated in drafting the manuscript; JNH selected the venues to be sampled; JLR and JNH carried out the field measurements. All authors read and approved the final manuscript.

## Pre-publication history

The pre-publication history for this paper can be accessed here:



## Figures and Tables

**Table 1:** 7 Downtown Boston bar/restaurants where air quality was measured. Smoking was permitted in the bar areas under the existing Boston regulations during the April 18, 2003 measurements, and was banned when the October 17, 2003 measurements were made. The monitors' inlets were ~1 m from the floor for all measurements.

**Venue^A^**	**Description**
**1. **Bar/Restaurant	A large "horseshoe" bar area dominates one large room. A small room opens out to the front. Bar caters to young singles clientele who gather after work. Food is also available but not central. Monitoring equipment was placed ~15 ft. from the bar against an outer wall in the bar area for both measurements.
**2. **Bar/Restaurant	A long rectangular bar dominates this famous bar/restaurant. One large open room. Wide variety of patrons from young singles, older couples and some tourists. Monitoring equipment was positioned against a wall ~6 ft. from one end of the bar and ~10 ft. from the front door in a virtually identical position for both measurements.
**3. **Bar/Restaurant	A large complex area dominated by a centrally located bar and stand-up eating area. This bar/restaurant is part of a chain well known for bar and traditional "pub-style" food. Patrons include both tourists and locals of diverse ages. On both occasions monitoring devices were placed in identical locations about 8 feet from the bar against a 5 ft. wall in the stand-up area.
**4. **Bar/Restaurant	A noisy and crowded venue. Patrons are almost exclusively 20 to 30 year old singles who gather from late afternoon to late at night. Bar food is available and served throughout both in the bar area and smaller dining room. Monitors were placed ~20 ft. from the bar against a windowed wall during the first (April visit), and against the bar for the return (October) visit.
**5. **Bar/Restaurant	A small, crowded, neighborhood bar/restaurant. The narrow bar area is ~15 ft. wide and ~40 ft. long with another ~20 ft. devoted to dining booths contiguous to the bar. Monitors were placed about 6 feet from the bar's middle against a wall in identical locations for each visit.
**6. **Bar/Restaurant	Grilled and sizzling-hot ethnic food is the main attraction of this bar/restaurant. The bar is contiguous to dining area #1, and ~10 ft. distant and open to dining area #2. Monitors were placed adjacent to tables in dining area #1 in April, and in dining area #2 in October.
**7. **Bar/Restaurant	Well-known upscale bar/restaurant chain frequented by both locals and tourists. The large rectangular raw shellfish bar area is separated from the main dining room by corridors but also has large dining tables encircling the bar. Monitors were placed against a wall adjacent to a dining table at ~12 ft. from the bar, and at adjacent tables for the two visits.
**8. **Hotel Room	11^th ^Floor Nonsmoking Rooms each visit, measurements made with open windows; hotel near Boston Garden Park.

^A^(Venue numbers are keyed to Figures 1 and 2.)

**Table 2:** April 18, 2003 Boston Indoor/Outdoor Pre-Ban Air Quality Survey Results

**Venue**	**Area (ft^2^)**	**Ceiling Ht. (ft)**	**Volume (m^3^)**	**Ave.^b ^# Persons Present (SD)**	**Ave.^b ^# Persons per 1000 ft**^**2**^	**Ave.^b ^# Burning Cigarettes (SD)**	**% of Persons Actively Smoking^b^**	**Estimated Smoker Prevalence % of all Persons**	**Ave.^b ^RSP, μg/m^3 ^(SD)**	**Ave.^b ^PPAH, ng/m^3 ^(SD)**	**D_s_, Active Smoker Density^a^**	**C_v_, Est.^c ^RSP Air changes per hour (h^-1^)**	**CO ppm (ave.) (SD)**	**CO_2 _ppm (Peak)**	**V_o _L/s-occ^g^**
**Pub #1**	1600	13	589	78.3 (11.2)	49	2.33 (0.58)	2.98	8.93	197 (55)	62 (23)	0.40	1.4	1.86 (0.13)	1100	7.9
**Pub #2**	4550	12.83	1653	131 (34)	29	0.5 (0.58)	1.5	1.15	43 (23)	6.4 (11.5)	0.03	0.75	1.90 (0.14)	680	29.1
**Pub #3**	5041	11	1570	111 (51.2)	22	3.67 (0.14)	3.3	9.9	57 (49)	38 (21)	0.23	3.74	2.08 (0.06)	800	15.3
**Pub #4**	1440	10	408	98 (2.7)	68	4.0 (1.73)	4.08	12.2	338 (120)	160 (59)	0.98	1.98	2.47 (0.21)	900	11.7
**Pub #5**	900	7.5	191	54 (1.4)	60	2.5 (0.71)	4.63	13.9	323 (113)	109 (68)	1.31	2.78	2.77 (0.33)	1480	5.0
**Pub #6**	2037	9.58	552	40.8 (9.25)	20	2.25 (0.5)	5.51	16.5	308 (80)	41.1 (68)	0.41	0.91	5.50 (1.05)	1150	7.4
**Pub #7**	1655	9	422	43.5 (2.1)	26	2.75 (0.5)	6.32	19.0	117 (39)	15.3 (9.0)	0.65	4.23	1.89 (0.07)	720	20.2
**Mean All**				**79.5 **(35.2)	**39 **(19.5)	**2.57 **(1.13)	**4.04 **(1.6)	**11.65 **(5.8)	**198 **(128)	**61.7 **(54.9)	**0.57 **(0.44)	**2.26 **(1.37)	**2.63 **(1.31)	**976 **(286)	**13.8 **(8.5)
**Mean all but # 6^d^**									**179 **(129)	**65.1 **(59.3)		**2.48 **(1.35)	**2.16 **(0.38)	**950 **(301)	**14.8 **(8.8)
**Hotel Rm**				1					6.45* (1.36)	2.81** (1.59)	0		**1.32 **(0.045)	**625 **(19)	
**Out-doors in transit **^f,h^				-					**18.6 **(11.7)	**15.8 **(11.7)	0		**2.14 **(0.45)	**473**^*e*^	

* 77 minute average (68 min before and 9 min after all Venue sampling); **73 minute average (65 min before, 8 min after sampling); (SD = standard deviations of measurments in parentheses; ^a^(D_s _in units of burning cigarettes per 100 m^3^);). ^c^(Using Habitual Smoker Model of Repace & Lowrey (1985):assumes 2 cigarettes per smoker-hour & 1.43 mg RS)P/cig: ETS-RSP = 650 D_s_/C_v_);^d^(excluding RSP and PPAH values from Pub #6). ^b^(Ave. of 3 measurements~ten minutes apart.) RH%: 25%–64%, mean 43.5% (9). T°C range: 12.7–20.9; mean 17.3 (2.3); ^e^(Average minimum background outdoor CO_2 _value). ^f^(average of all outdoor measurements); ^g^(assumes C_o _= 473 ppm), ^h^(On sidewalks; crossing streets).

**Table 3:** October 17, 2003 Boston Indoor/Outdoor Air Quality Survey Smoke-Free Results Post-Ban

**Venue**	**Area (ft^2^)**	**Ceiling Ht. (ft)**	**Volume (m^3^)**	**Ave. ^b ^Persons Present (SD)**	**Ave.^b ^Persons per 1000 ft**^2^	**Ave.^b ^RSP, μg/m^3 ^(SD)**	**% of Pre-ban RSP Level**	**Ave.^b ^PPAH, ng/m^3 ^(SD)**	**% of Pre-ban PPAH Level**	**CO ppm (ave.) (SD)**	**CO_2 _ppm (peak)**	**V_0_, L/s-occ^d,f,g^**
**Pub #1**	1600	13	589	54.6 (1.15)	34	7.47 (1.46)	3.8	8.56 (4.99)	13.8	1.04 (0.084)	950	10.8
**Pub #2**	4550	12.83	1653	99.3 (26)	21.8	16.3 (4.75)	38	1.61 (2.14)	25.2	2.89 (0.37)	900	12.1
**Pub #3**	5041	11	1570	123 (20.6)	24.4	1.39 (1.44)	2.4	5.98 (13.7)	15.7	1.30 (0.24)	800	16.0
**Pub #4**	1440	10	408	92.7 (22.5)	64.4	6.26 (1.05)	1.9	12.2 (5.13)	7.6	0.82 (0.18)	950	10.8
**Pub #5**	900	7.5	191	69 (1.73)	76.7	13.5 (3.16)	4.2	7.45 (4.00)	6.8	0.92 (0.09)	940	11.0
**Pub #6**	2037	9.58	552	50.3 (2.08)	24.7	525 (274)	170	1.55 (3.82)	3.8	7.94 (1.48)	1260	6.5
**Pub #7**	1655	9	422	48.3 (11.0)	29.0	1.49 (0.96)	1.2	2.14 (1.24)	14.0	0.48 (0.19)	720	21.5
**Mean all Venues**				**76.7 **(28.8)	**39 **(22)	**81.6 **(196)	**41**	**5.64 **(4.09)	**9.1**	**2.20 **(2.64)	**931 **(169)	**12.7 **(4.78)
**Mean all but # 6^a^**						**7.73 **(6.13)	**4.3**	**6.32 **(4.02)	**10.2**	**1.24 **(0.85)	**877 **(96)	**13.7 **(4.3)
**Non-smoking Hotel Room**				1		2.14* (1.16)	**33**	2.42** (1.54)	**86**	**0.56 **(0.037)	**573 **(44)	
**Outdoors/In Transit^h^**						**7.82**^*c*^	**42**	**9.05**^*c*^	**57**	**1.32**	**487**^*e*^	

(SD = standard deviations of measurements in parentheses); *(91 min Ave., 68 min before venues, 23 min after); **(85 min Ave., 65 min before venues, 20 min after; ^a^(excluding Pub #6). ^b^Ave. of 3 measurements~ten min apart;); ^c^(Time-weighted mean). ^d^(based on ASHRAE 62 formula); ^e^(average minimum background); ^f^(assumes C_o _= 487 ppm);^g^(assumes C_o _= 487 ppm); ^h^(On sidewalks; crossing streets). Range in air temperature: 17.5 – 21.8°C, mean 19.8°C; range in relative humidity: 28%–48%, mean 38%.

**Table 4:** Levels of fine particulate (PM_2.5_) air pollution in units of micrograms per cubic meter ((μg/m^3^) and corresponding U.S. health advisory descriptors with accompanying simplified color code (USEPA, 1999).

**PM_2.5 _(μg/m^3^) AQI Break-points**	**Air Quality Index**	**Category**	**Color Code**
0.0 – 15.4	0 – 50	Good	Green
15.5 – 40.4	51 – 100	Moderate	Yellow
40.5 – 65.4	101 – 150	Unhealthy SG*	Orange
65.5 – 150.4	151 – 200	Unhealthy	Red
150.5 – 250.4	201 – 300	Very unhealthy	Violet
250.5 – 350.4	301 – 400	Hazardous	Maroon
350.5 – 500.4	401 – 500	Very Hazardous	Maroon
> 505	500	(Significant Harm)	**

*SG = sensitive groups; **exists, but is not a part of the AQI as outdoor air never gets this polluted due to federal and state regulation and enforcement action (Ellsworth, 2005).

**Table 5:** Comparison of Pre-and-Post Ban RSP Levels with the Federal AQI.

**1. Venue**	**2. Pre-Ban Ave. RSP, μg/m^3^**	**3. Potential AQI Designation**	**4. Est. SHS-RSP (RSP_pre-ban _– RSP_post-ban_)**	**5. Junker Irritation Ratio^a^**	**6. Junker Odor Threshold Ratio^b^**
Pub #1	197	VERY UNHEALTHY	188	43	188
Pub #2	43	UNHEALTHY SENSITIVE GROUPS	119	27	119
Pub #3	57	UNHEALTHY SENSITIVE GROUPS	57	13	57
Pub #4	338	HAZARDOUS	332	75	332
Pub #5	323	HAZARDOUS	309	70	309
Pub #6	308	HAZARDOUS	--	--	--
Pub #7	117	UNHEALTHY	116	26	116
Mean All Venues	198	VERY UNHEALTHY	--	--	--
Mean all but # 6	179	VERY UNHEALTHY	171	39	171
Non-smoking Hotel Room	6.45	GOOD	NA	NA	NA
Outdoors/In Transit	18.6	MODERATE	NA	NA	NA

^a ^(Ratio of SHS-RSP in Col. 4 to Junker Irritation Threshold of 4.4 μg/m^3^). ^b^(Ratio of SHS-RSP in Col. 4 to Junker Odor Threshold of 1 μg/m^3^). NA = not applicable.

## References

[B1] National Research Council (1986). Environmental tobacco smoke – measuring exposures and assessing health effects.

[B2] Surgeon General (2006). The Health Consequences of Involuntary Exposure to Tobacco Smoke, A Report of the Surgeon General.

[B3] NIOSH Current Intelligence Bulletin #54 (1991). Environmental Tobacco Smoke in the Workplace, Lung Cancer and Other Health Effects.

[B4] U.S. EPA, Health Effects of Passive Smoking (1992). Assessment of Lung Cancer in Adults, and Respiratory Disorders in Children EPA/600/6-90/006F.

[B5] OSHA US Dept of Labor, Occupational Safety & Health Administration 29 CFR Parts 1915, 1926, and 1928 Indoor air quality, proposed rule Fed Reg 59 # 65, Tues April 5, 1994, 15968-16039.

[B6] CALIFORNIA ENVIRONMENTAL PROTECTION AGENCY (2005). Health Effects of Exposure to Environmental Tobacco Smoke, SRP-Approved.

[B7] National Toxicology Program (2000). 9th Report on Carcinogens. US Dept of Health & Human Services, National Institute of Environmental Health Sciences, Research Triangle Park, NC.

[B8] Hoffmann D, Hoffmann I, O'Neill I, Brunnemann K, Dodet B, Hoffmann D (1987). Significance of exposure to sidestream tobacco smoke. Ch. 1. IARC Scientific Publications no81, Environmental Carcinogens – Selected Methods of Analysis – Volume 9 Passive Smoking.

[B9] Repace JL, Ott WR, Wallace LA, Steinemann AM (2006). Human Exposure to Secondhand Smoke. Human Exposure Analysis.

[B10] Pope CA, Dockery DW (2006). Health effects of fine particulate air pollution: lines that connect. 2006 Critical Review. J Air & Waste Manage Assoc.

[B11] (2004). IARC Monographs on the Evaluation of Carcinogenic Risks to Humans Tobacco Smoke and Involuntary Smoking.

[B12] Repace JL (2004). Respirable Particles and Carcinogens in the Air of Delaware Hospitality Venues Before and After a Smoking Ban. Journal of Occupational and Environmental Medicine.

[B13] American Society of Heating Refrigerating and Air Conditioning Engineers (1989). Ventilation for Acceptable Indoor Air Quality, ASHRAE Standard 62-1989, Atlanta, GA.

[B14] Repace JL, Lowrey AH (1980). Indoor Air Pollution, Tobacco Smoke, and Public Health. SCIENCE.

[B15] Ott WR (1999). Mathematical models for predicting indoor air quality from smoking activity. Environmental Health Perspectives.

[B16] Weather Underground. http://www.wunderground.com.

[B17] Connally GN, Carpenter C, Alpert HR, Skeer M, Travers M (2005). Evaluation of the Massachusetts Smoke-free Workplace Law, a preliminary report, Harvard School of Public Health, Tobacco Research Program. http://www.hsph.harvard.edu/php/pri/tcrtp/Smoke-free_Workplace.pdf.

[B18] Levy JI, Bennett DH, Melly SJ, Spengler JD (2003). Influence of traffic patterns on particulate matter and polycyclic aromatic hydrocarbon concentrations in Roxbury, Massachusetts. J Exposure Analysis & Environmental Epidemiology.

[B19] Federal Register: July 18, 1997.

[B20] Ware JM [EDITORIAL] Particulate Air Pollution and Mortality – Clearing the Air. New England J Medicine.

[B21] Wallace L (1996). Indoor particles: a review. J Air & Waste Mgt Assoc.

[B22] US Environmental Protection Agency. http://www.epa.gov/airtrends/pm.html.

[B23] Mulcahy M, Evans DS, Hammond SK, Repace JL, Byrne M (2005). Secondhand smoke exposure and risk following the Irish smoking ban: an assessment of salivary cotinine concentrations in hotel workers and air nicotine levels in bars. Tobacco Control.

[B24] MMWR Prevalence of Current Cigarette Smoking Among Adults and Changes in Prevalence of Current and Some Day Smoking – United States, 1996-2001. MMWR.

[B25] Weber A, Grandjean E, O'Neill IK, Brunnemann KD, Dodet B, Hoffmann D (1987). Acute effects of environmental tobacco smoke. IARC Scientific Publications no81, Environmental Carcinogens – Selected Methods of Analysis – Volume 9 Passive Smoking.

[B26] Junker MH, Danuser B, Monn C, Koller T (2001). Acute sensory responses of nonsmokers at very low environmental tobacco smoke concentrations in controlled laboratory settings. Environ Health Perspectives.

[B27] Repace JL, Jinot J, Bayard S, Emmons K, Hammond SK (1998). Air nicotine and saliva cotinine as indicators of passive smoking exposure and risk. Risk Analysis.

[B28] Travis CC, Richter SA, Crouch EAC, Wilson R, Klema ED (1990). Cancer Risk Management. Environmental Science and TEchnology.

[B29] Biener L, Fitzgerald G (1999). Smoky bars and restaurants: who avoids them and why?. J Public Health Management and Practice.

[B30] Abrams SM, Mahoney MC, Andrew Hyland A, Cummings KM, Davis D, Song L (2006). Early Evidence on the Effectiveness of Clean Indoor Air Legislation in New York State. Am J Public Health.

[B31] ATSDR Agency for Toxic Substances and Disease Registry. ToxFAQs for Polycyclic Aromatic Hydrocarbons (PAHs).

[B32] Glantz SA, Parmley WW (1991). Passive smoking and heart disease. Circulation.

[B33] Biener L, Harris JE, Hamilton W Impact of the Massachusetts tobacco control programme: population based trend analysis. BMJ.

[B34] (2003). Massachusetts Department of Environmental Protection.

[B35] U.S. EPA Office of Air and Radiation EPA's Revised Particulate Matter Standards. Fact Sheet, Office of Air Quality Planning & Standards.

[B36] (1996). ASHRAE Handbook 1996 HVAC Systems and Equipment.

[B37] Edwards R, Hasselholdt CP, Hargreaves K, Probert C, Holford R, Hart J, Van Tongeren M, Watson AFR (2006). Levels of second hand smoke in pubs and bars by deprivation and food-serving status: a cross-sectional study from North West England. BMC Public Health.

[B38] Ott W, Switzer P, Robinson J (1996). Particle concentrations inside a tavern before and after prohibition of smoking: evaluating the performance of an indoor air quality model. J Air & Waste Manage Assoc.

[B39] Travers MJ, Cummings KM, Hyland A, Repace J, Babb S, Pechacek T, Caraballo R (2003). Indoor Air Quality in Hospitality Venues Before and After Implementation of a Clean Indoor Air Law – Western New York. MMWR.

